# Dual Functions of T Lymphocytes in Breast Carcinoma: From Immune Protection to Orchestrating Tumor Progression and Metastasis

**DOI:** 10.3390/cancers15194771

**Published:** 2023-09-28

**Authors:** Mohammadrasul Zareinejad, Fereshteh Mehdipour, Mina Roshan-Zamir, Zahra Faghih, Abbas Ghaderi

**Affiliations:** Shiraz Institute for Cancer Research, School of Medicine, Shiraz University of Medical Sciences, Shiraz 71348-45505, Iran; m.rasul.z.nejad@gmail.com (M.Z.); mehdipourf@sums.ac.ir (F.M.); minarzs95@gmail.com (M.R.-Z.)

**Keywords:** breast cancer, immune system, T lymphocyte, helper subset, cytotoxic subset, regulatory subset

## Abstract

**Simple Summary:**

New insights into the foundation of cellular and molecular cancer immunology have revealed that immune cells play crucial roles in the development and growth of breast cancer (BC). T-cells are one of the most important cells in the tumor microenvironment and are divided into several subtypes including helper, cytotoxic, and regulatory subsets according to their transcription factors, markers, and functions. This article provides a comprehensive review of contradictory functions of various T-cell subsets in the prognosis and treatment of patients with BC, and crosstalk between tumor cells and T-cells. The literature shows that the role of T-cells in BC immunity depends on a variety of factors, including the tumor type or subtype, the stage of the disease, the localization of the cells in the tumor tissue and the presence of different cells or cytokines.

**Abstract:**

Breast cancer (BC) is the most common cancer type in women and the second leading cause of death. Despite recent advances, the mortality rate of BC is still high, highlighting a need to develop new treatment strategies including the modulation of the immune system and immunotherapies. In this regard, understanding the complex function of the involved immune cells and their crosstalk with tumor cells is of great importance. T-cells are recognized as the most important cells in the tumor microenvironment and are divided into several subtypes including helper, cytotoxic, and regulatory T-cells according to their transcription factors, markers, and functions. This article attempts to provide a comprehensive review of the role of T-cell subsets in the prognosis and treatment of patients with BC, and crosstalk between tumor cells and T-cells. The literature overwhelmingly contains controversial findings mainly due to the plasticity of T-cell subsets within the inflammatory conditions and the use of different panels for their phenotyping. However, investigating the role of T-cells in BC immunity depends on a variety of factors including tumor types or subtypes, the stage of the disease, the localization of the cells in the tumor tissue and the presence of different cells or cytokines.

## 1. Introduction

Breast cancer (BC) is a clinically and histologically heterogeneous disease, consisting of different subtypes with various prognoses [1]. Its incidence, mortality, and survival rates vary among different ethnicities and populations and depend on genetic and environmental factors, lifestyle, and population structure [2]. Despite recent advances in BC treatment, its mortality rates continue to rise especially in developing countries, highlighting the need for developing new treatment strategies including targeting driver mutations and modulation of the immune system [3].

New insights into the cellular and molecular mechanisms involved in cancer development have revealed that immune cells play crucial roles in the development and growth of BC as well. With their dual functions, immune cells can produce a pro-tumorigenic inflammatory environment on one hand, and cause tumor rejection on the other. Since the effect of an immune response is largely determined by the type of stimulated immune response, understanding the crosstalk between tumor cells and the cells of the immune system is of great importance. In this regard, analyses of the total BC tumor tissue have detected a broad spectrum of genes related to immune responses, reflecting both innate and adaptive immunity including T-cell metagene (a set of related or co-regulated genes with similar expression patterns, functions, and regulatory elements) [4], B-cell metagene [5,6], or related signaling pathways [7]. Consistent with genetic studies, phenotyping investigations have also indicated an increase in leukocyte infiltration along with the tumor growth [8]. The composition of infiltrating lymphocytes is very heterogeneous and includes T-cells, both CD4^+^ and CD8^+^ subtypes, and to a lower extent, B-cells, macrophages, and NK cells [8,9]. Although there is no agreement on the specific immune cell subset, generally, an immune-enriched signature is considered an indicator of an active anti-tumor immune response and better clinical outcomes [10]. However, some studies reported the association of higher numbers of tumor-infiltrating lymphocytes (TILs) subpopulations with aggressive phenotypes of the disease [11,12]. However, this could be a primary consequence of the immune response to the tumor growth; it also indicates that the role of immune cells in the BC prognosis, progress, and/or response to treatment is still controversial. Besides different molecular subtypes of BC which have different behaviors [13], one of the most important reasons for the discrepancy and lack of coordination among the studies is that only one or a few major cell types (i.e., CD3^+^) have been investigated regardless of their functional (stimulatory or inhibitory) subgroups.

**Figure 1:**
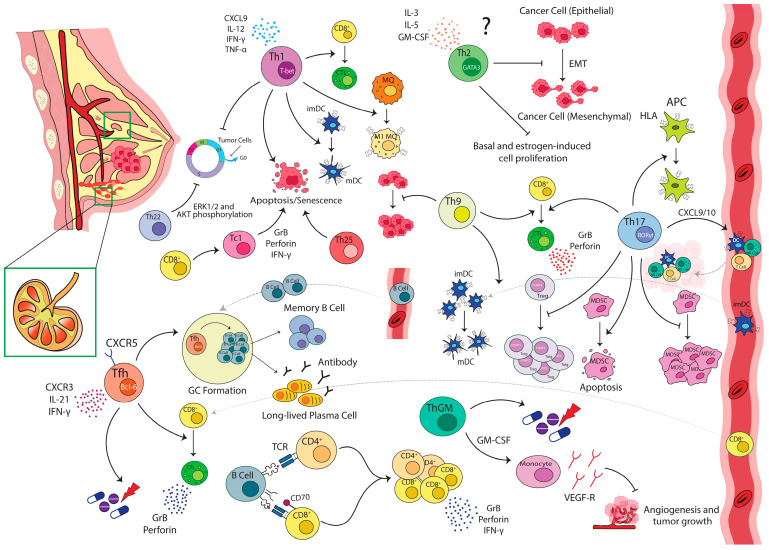
**Anti-tumor functions of T-cell subsets in breast cancer.** Through the secretion of inflammatory mediators, i.e., IFNγ and TNFα, **Th1** arrests the cell cycle in G1/G0 and induces apoptosis and senescence in the breast tumor cells. Besides direct effects, Th1 increases antigen presentation, proliferation and cytolytic function of CTLs, activates M1-macrophages and leads to maturation of DCs. Intriguingly, there are some reports that TSLP-stimulated **Th2** could reprogram tumor cells to terminal differentiation, and directly block carcinogenesis and EMT through secreting cytokines, i.e., IL-3, IL-5, and GM-CSF. It could also inhibit basal and estrogen-induced cell proliferation. **Th17** produces anti-tumor chemokines, attracts and stimulates NK and DCs, induces MHC-I and II expression, decreases proliferation and induces apoptosis in MDSCs. It also enhances CTL activity and crosstalk with Th1 but reciprocally regulates Tregs. **Th22** reduces tumor growth and promotes cell cycle arrest via the reduction of ERK1/2 and AKT phosphorylation. **Th25** induces apoptosis in tumor cells. Besides the direct inhibitory effect on tumor cells, **IL-9** and IL-9-producing T-cells promote tumor-specific T-cell responses, particularly CTLs, through the activation, survival and secretion of granzyme B, perforin and IFN-γ from T-cells. In addition, they recruit leukocytes including DCs into tumor tissues, increase their survival, and enhance antigen-presentation in the tumor draining lymph nodes. **ThGM** inhibits tumor progression and metastasis through its effects on monocytes and secretion of soluble VEGFR-1, which in turn inactivates the VEGF and blocks angiogenesis. In addition, GM-CSF overexpressing cells exert more sensitivity to anti-cancer drugs. **Tfh** is the main source of CXCL13 in breast tumors, directing B-cells and promoting lymphoid structure and GCs formation. It constitutes one of the important components of GCs in both draining lymph nodes and tertiary lymphoid structures (TLS) in tumor bed, where it provides help for production of immunoglobulins, and induces effector memory B-cells and plasma cells. It also regulates the activation and recruitment of CTLs. On the other side, B-cells could present or cross-present antigens to CD4^+^ and CD8^+^ T-cells. Engagement of CTLs with bystander B-cells through CD27/CD70 contact also leads to the proliferation and survival of CD8^+^ T-cells. **Abbreviations:** APC: antigen-presenting cell, CTL: cytotoxic T-cells, DC: dendritic cells, EMT: epithelial–mesenchymal transition, GC: germinal center, NK: natural killer, MDSC: myeloid-derived suppressor cells, Th: T helper, Tfh: T follicular helper, Treg: regulatory T-cells, TSLP: thymic stromal lymphopoietin, VEGF-R: vascular endothelial growth factor receptor.

**Figure 2:**
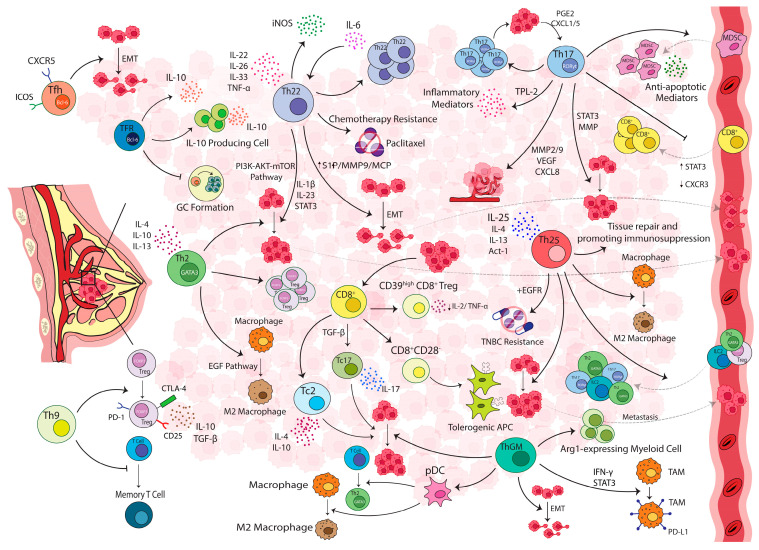
**Pro-tumor functions of T-cell subsets in breast cancer**. **Th2** increases the frequency of Tregs and induces M2-macrophage differentiation and subsequently promotes tumor survival, growth, and metastases. **Th17** regulates angiogenesis, induces pro-invasive factors (i.e., IL-17, IL-22, and IL-23, MMPs) and promotes the proliferation, survival, and invasion of malignant cells. It could also interfere with CTLs migration through the activation of STAT3 signaling, and reduction of CXCR3 expression. IL-17 supports inflammation in the tumor microenvironment indirectly by inducing TPL2, and promotes the recruitment, suppressive functions, and release of anti-apoptotic factors from MDSCs. **Th22** enhances tumor cells migration by activating the STAT3/MAPKs/AKT pathway. It also stimulates S1P production in MSCs and promotes the chemotactic migration of breast tumor cells and their metastasis to bone marrow. IL-22 accelerates this process by increasing MCP-1 and MMP-9 in MSCs. In addition, following the upregulation of IL-22, the PI3K-AKT-mTOR pathway is activated and increases the growth, migration, and invasion of the tumor cells. It also renders them resistant to anti-mitotic and anti-EGFR therapeutic agents. **Th25** promotes immunosuppression through the recruitment and activation of type 2 immune cells (Th2 and M2-macrophages). **ThGM** increases the expression of PD-L1 on TAMs via an IFN-γ/STAT3-dependent pathway and induces an EMT/stemness-like invasive phenotype or accumulates Arginase 1 expressing myeloid cells. It also induces pDC, which in turn deviates naive CD4^+^ T-cells and macrophages into a pro-tumorgenic Th2 and pro-invasive TAM-like phenotype, respectively. **Th9** enhances the immunosuppressive function of Tregs, prevents immunological memory formation, and promotes the development of hematological malignancies. **Tfh** plays a role in the regulation of EMT during lymph node metastasis. **TFR** controls Tfh and GC responses and prevents antibody production. It also increases IL-10 directly or indirectly via the differentiation of IL-10-producing B-cells. **CD8^+^ T-cells** might differentiate into pro-tumor and regulatory cells (CD39^high^CD8^+^ Tregs, CD8^+^CD28^−^ T-cells, Tc2, and Tc17 cells) and promote tumor growth. **Abbreviations:** Arg1: arginase 1, APC: antigen-presenting cell, CTL: cytotoxic T-cells, DC: dendritic cells, EGF: epidermal growth factor, EGFR: epidermal growth factor receptor EMT: epithelial–mesenchymal transition, iNOS: inducible nitric oxide synthase, GC: germinal center, NK: natural killer, MDSC: myeloid-derived suppressor cells, MMP: matrix metalloproteinases, MCP: monocyte chemoattractant protein, pDC: plasmacytoid dendritic cells, PGE2: prostaglandin E2, S1P: sphingosine-1-phosphate, Th: T helper, Tfh: T follicular helper, TFR: T follicular regulatory, Treg: regulatory T-cells, TPL2: tumor progression locus 2, TSLP: thymic stromal lymphopoietin, TAMs: tumor-associated macrophages, TNBC: triple-negative breast cancer, VEGF-R: vascular endothelial growth factor receptor.

The specific influences of individual immune subsets in the breast tumor microenvironment have been addressed in several studies. Their results generally indicate a mixture of activation and suppression in TILs and suggest that their position and prevalence within the tumor dictate responsiveness. In many of these studies, CD3^+^ T-cells, as critical regulators of adaptive immune responses, were reported as the major leukocyte population detected in the breast tumor tissue. Among various T-cell subsets, CD4^+^ T-cells (in some cases CD8^+^ lymphocytes) are more prevalent in the tumor microenvironment and peripheral blood [14,15,16]. In most cases, T-cells (both CD4^+^ and CD8^+^) in the tumor microenvironment display an activated phenotype represented by an increased expression of activation markers (i.e., CD69, CD25, CD95, CD44, and HLA-DR) and concomitant decrease in naive T-cells markers (CD45RA and CCR7). The activation markers, however, do not necessarily mean that intratumoral T-cells are fully functional [17,18]. In line with this, recent molecular profiling studies demonstrate that infiltrating CD4^+^ T-cells are effector memory cells, containing all helper subpopulations including Th1, Th2, Th17, Tfh, and regulatory T-cells (Tregs) [19]. However, they represent a restricted repertoire of receptors [20], helper cytokines and chemokines, implying suboptimal activation levels [19]. The complexity of CD4^+^ T lymphocytes (presumably Th2 or Tregs or both) in conjunction with CD8^+^ T lymphocytes and CD68^+^ tumor-associated macrophages (TAMs) were also reported that could be predictive of overall survival (OS) and relapse-free survival (RFS) in node-positive human BC [19,21]. These results, in addition to our observations, indicated that although there are no significant differences in the major populations of lymphocytes (CD3^+^ T-cell, and its main subgroups, CD4^+^ helper and CD8^+^ cytotoxic, or B-cells), with the progress of the disease, functional subgroups of CD4^+^ and CD8^+^ T lymphocytes show significant variations [22,23]. This confirms that the investigation of general markers, like CD4 and CD8, alone does not fully represent their functional status. Therefore, a comprehensive effort is needed to investigate the main components of the adaptive immune system involved in tumor growth, both effectors and regulators, in BC patients with different pathological properties. In what follows, we reviewed the available literature and highlighted significant recent discoveries demonstrating the contradictory functions of various T-cell subsets as key regulators of immune responses during BC growth and progression (Figure 1 and Figure 2).

## 2. Th1/Th2 Paradigm in Breast Carcinoma

In tumor immunology, it is generally believed that Th1 cells are often critical components in anti-tumor immune responses due to their ability to produce IFN-γ, activate macrophages, and boost the killer CD8^+^ T-cells, whereas type 2 helper responses can promote cancer development or metastasis [24]. Clinical evaluation of human BCs also showed the presence of Th2 lymphocytes accompanied by an increase in the frequency of Tregs during cancer development. In this regard, the ratio of Th2/Th1 cells in primary tumors as well as sentinel lymph nodes (SLNs), where Th2 cells are more frequent than Th1 cells, has been shown to be positively correlated with tumor stage, metastatic lymph nodes, larger tumor size, and reduced OS [17,25,26,27]. In addition, it was shown that the cellular immune responses, from dendritic cell (DC) maturation to Th1 responses, were also less active in SLNs than in non-SLNs in these patients before metastasis. It has been suggested that as the tumor grows, immunosuppressive secretions from the tumor are drained into the SLNs and change the immune responses in both the tumor site and draining LNs in favor of a reduced Th1/Th2 ratio. These results support the idea that changes in the immune profile of SLNs can provide a niche for tumor LN metastases, which in turn accelerates further tumor growth and spreading. After metastasis, DC maturation was found to be triggered and followed by the upregulation of Th1 responses, which could be a reflection of antigen-specific priming in SLNs; however, Th2 and Tregs responses upregulated in parallel [28]. Immunophenotyping analysis of the intracellular IFN-γ and IL-4 also indicated a shift toward the Th2 phenotype in whole blood. The secretion of IFN-γ was strongly impaired in BC, whereas the levels of TNF-α and IL-1β were comparable with those obtained from normal subjects [29]. In line with these findings, our results confirmed an increased accumulation of Th2 and Tregs in the breast tumor-draining lymph nodes (TDLNs) of patients with at least one involved lymph node (LN^+^ patients). Conversely, a decrease in IFN-γ production was observed with tumor progression from stage I to stage III. The expression intensity of IFN-γ also showed a strong correlation with the frequency of IFN-γ-producing Th1 cells, indicating that the activity of the Th1 cells decreases along with the decline in their numbers [22]. Overall, these findings indicate that metastasis might be accelerated by inflammatory Th2 responses along with the reduction in Th1 responses. Similarly, Fracol et al. showed a reduction in anti-HER-3 IFN-γ immunity during breast tumorigenesis, highlighting the important role of the Th1 cells in surveilling tissues against overexpressed antigens. Tumor recurrence and incomplete response to neoadjuvant therapy also correlated with the suppression of Th1 immune responses, and accordingly, Th1 status could be introduced as a prognostic factor in patients with invasive BC [30].

Gene expression analysis further confirmed the results of the clinical studies. While a decrease in Th1-related genes in tumor tissues compared with the controls was observed in BC [31], unsupervised profiling of BC stroma demonstrated that a gene signature functionally enriched in Th1-type immune response elements is associated with favorable prognosis (>98%, 5-year survival) [32]. Consistently, an IFN-based gene signature has also been detected in triple-negative patients who are more likely to remain metastasis-free and independently predicted improved RFS and OS [33,34]. Similarly, the gene expression profiling of purified CD4^+^ T-cells from primary tumors, axillary LNs, and peripheral blood of BC patients suggested that a Th1 signature including 12 genes, e.g., chemokine (C-X-C motif) ligand 9 (CXCL9) and IFN-γ, predicts better survival in the human epidermal growth factor receptor 2 (HER2^+^) BC subtype [19]. These observations imply that a tumor environment enriched in type 1 cytokines would result in an increased antigen presentation, proliferation, and cytolytic function of cytotoxic T-cells (CTLs) on one hand, and PD-L1 expression, growth arrest in G_1_/G_0_, HER2 oncogene inactivation, on the other hand, and consequently the apoptosis and senescence of the breast tumor [19,35,36,37,38]. Consistently, the response to HER2-targeted therapies has been correlated with the presence of Th1 immunity, which increases MHC-I expression and promotes tumor cell recognition by CTLs and cytolysis [36,39]. Additionally, it has been shown that the presence of Th1-mediated immunity increases the efficiency of BC treatments [36]. Accordingly, the majority of immunotherapies aim to restore Th1 immunity and shift Th2 toward Th1 cell response [25,40].

In line with these findings from human tumors, studies on transgenic mouse models of aggressive mammary adenocarcinoma demonstrated that the elimination of endogenous T-cells significantly reduced pulmonary metastases with no effects on developing primary tumors. Further studies demonstrated that metastasis specifically depended on CD4^+^ T-cells, as their absence reduced the metastasis rate. They found that CD4^+^ T-cells expressing high levels of IL-10, IL-4, and IL-13 compared with those expressing IFNγ or IL-17 enhanced pulmonary metastasis indirectly through the induction of M2-macrophages. M2 macrophages increase the invasive behavior of malignant mammary epithelial cells by promoting intracellular signaling cascades such as the epidermal growth factor (EGF) pathway. This phenomenon was independent of cytotoxic T-cells, indicating that pro-tumor activity of CD4^+^ T-cells did not involve the suppression of CTLs [41]. Spontaneous breast carcinomas also developed more quickly in HER2/neu transgenic mice when T-cells were depleted. However, it could be considered as evidence for slowing tumor growth through T-cell-mediated immunosurveillance; yet, the blockade of IL-13 further enhanced this effect, which confirmed a role for the type 2 immune response in promoting tumor growth [42]. Assessing the anti-tumor and biological activities of endogenous type 1 and type 2 effector T-cell subpopulations at the primary site of the mammary tumor also revealed differential infiltration kinetics of effector T-cell subpopulations at the tumor site along with a general delay in CD3^+^ T-cells infiltration. Cytokine profiling showed elevated IL-4-producing Th2 cells earlier than IFN-γ-producing Th1 cells, which remained noticeably higher and was linked with tumor growth and metastases. It suggested that this initial increase in IL-4-producing Th2 cells might antagonize and/or postpone the emergence of a more favorable Th1 immune response in untreated animals. As a result, infiltrating lymphocytes would experience immunological ignorance and/or anergy, which would ultimately encourage the development and proliferation of the tumor [18]. As revealed by in vitro studies, tumor growth could be directly enhanced by IL-4 and IL-13 through the activation of their receptors on epithelial cells [43]. Consistently, intense IL-13 staining in breast TILs was observed in tumor cells which, along with the expression of phosphorylated signal transducer and activator of transcription-6 (pSTAT6), suggested that IL-13, in fact, delivers growth signals to cancer cells. It seems that BC educates DC cells in a manner to induce IL-13 secretion by Th2 lymphocytes, and facilitates their development [44]. IL-4 also promoted tumor cell survival by making them resistant to apoptosis [45]. It was also shown that BC cell-derived thymic stromal lymphopoietin (TSLP) fosters an inflammatory Th2 microenvironment by prompting OX40L expression on DCs. Antibodies neutralizing TSLP or OX40L inhibited IL-13 production and tumor growth in a xenograft model [46]. However, Boieri et al. proposed an opposite role for Th2 cells in BC, as they showed that Th2 cells stimulated by TSLP could reprogram tumor cells and induce their terminal differentiation. They could also directly block carcinogenesis and Epithelial-to-Mesenchymal Transition (EMT) through the secretion of cytokines such as IL-3, IL-5, and GM-CSF [47]. There are also reports on human breast carcinoma cell lines, particularly estrogen receptor (ER) α-expressing lines, showing that IL-4 and IL-13 could inhibit basal and estrogen-induced cell proliferation in vitro, and in xenograft models [48,49]. In addition, it was also shown that baseline IL-4/13 signaling is implicated in normal mammary gland development [50]. Epidemiological studies also implied less susceptibility to BC in patients with allergic diseases, a phenomenon believed to be mediated by inflammatory Th2 cells [51,52]. These findings suggest a more complicated role for Th2 cells in BC influenced by the threshold of activation, along with the complexity, plasticity, or the involvement of new subsets sharing effector molecules, i.e., ThGM or Th25 (discussed later), as well.

## 3. Th17 in Breast Carcinoma

Th17 cells constitute the third subset of effector T helper cells with a potent inflammatory nature, characterized by their distinctive cytokine, IL-17A [53]. Although the contribution of Th17 and its related molecules in infection and autoimmunity is well-documented, its role in tumor immunity remains elusive. The tumor-infiltrating Th17 cells are reported in various cancers such as BC with both pro- and anti-tumor properties. In most of these studies, IL17-producing T-cells have been associated with disease progression, worse prognosis, triple-negative molecular subtypes, shorter disease-free survival (DFS), and genes related to the tumors’ proliferation and survival [54,55,56]. The pro-tumor properties of Th17 cells in BC (Figure 2) are attributed to multiple mechanisms, including the regulation of angiogenesis, induction of pro-invasive factors (i.e., IL-17, IL-22, and IL-23), metalloproteinases (MMPs) that promote proliferation, survival, and the invasion of malignant cells, interfering with CD8^+^ T-cell migration through the activation of STAT3 signaling and subsequent reduction of CXCR3 expression [57,58,59,60]. The role of IL-17 in angiogenesis is enhanced through the induction of angiogenic factors such as MMP-2, MMP-9, vascular endothelial growth factor (VEGF), and CXCL8 [61]. IL-17 also supports inflammation in the tumor microenvironment indirectly by inducing tumor progression locus 2 (TPL2) [62].

The data also indicate that tumor cells, tumor-associated fibroblasts, and myeloid-derived suppressor cells (MDSCs) not only produce the chemokines mediating Th17 recruitment but also create a pro-inflammatory milieu and a cell–cell contact framework that enhance Th17 cell differentiation and expansion in the breast tumor microenvironment. Accordingly, its neutralization could lead to the reduction of tumor growth and migration of tumor cells to the secondary tumor site [54,61,63,64,65]. Direct interaction of CD40 on MDA-MB231 cells with CD40L on T-cells was shown to upregulate Transforming Growth Factor-beta (TGF-β), induce Th17 differentiation, and increase the proliferation of tumor cells via STAT-3 signaling [66]. It was also shown that tumor cells through secretion of some mediators, i.e., Prostaglandin E2 (PGE2) and CXCL1/5, led to Th17 expansion and CXCL1 production, and subsequently BC growth and development [63,64]. On the other hand, the IL-17 response was found to promote recruitment, suppressive functions, and the release of anti-apoptotic factors from MDSCs [61,67]. In contrast, an anti-tumor role for Th17 cells and their related molecules has been suggested by independent groups, including us, through the observation of negative correlations of the frequency of Th17 cells with the size and stage of BC [22,68] or longer survival [69]. In this regard, we observed that the mean expression of IL-17 in Th17 cells declined significantly in node-positive patients, and in those in higher stages [22]. Yang et al. also demonstrated an association between the frequency of Th17 lymphocytes and a more favorable prognosis in BC. Nearly all cancerous tissue specimens showed an increased infiltration of Th17 cells compared with the normal breast tissues. A negative association was also observed between the prevalence of Th17 cells and TNM-stage, the invasion of the blood vessel, and higher numbers of metastatic LNs [68]. A bioinformatics analysis also indicated an association between the Th17 metagene and good prognosis and longer survival in Triple-negative BC (TNBC) patients [69]. In another study, it was shown that the accumulation of both Th17 and Tregs in the breast tumor microenvironment occurred at the early stage of the disease; however, with tumor progression, Th17 infiltration gradually decreased, while Treg infiltration increased and resulted in Treg dominance in advanced stages of BC [70]. In addition, the frequency of Th17 in peripheral blood was lower in HER2^+^ patients compared with the healthy ones, while it increased following a treatment with Trastuzumab. It also had an inverse correlation with Tregs in metastatic patients [71]. The anti-tumor activities of Th17 could be attributed to multiple mechanisms (Figure 1), indirectly through fighting bacterial and viral infections, known to play a significant role in the pathogenesis of many cancers, or directly through the regulation of Tregs in a reciprocal manner, the induction of MHC-I and II expression, the enhancement of CTL activity, and crosstalk with Th1 [57,72]. IL-17 produced by Th17 cells was shown to synergize with IFN-γ in the induction of anti-tumor chemokines CXCL9 and CXCL10 that attract and stimulate NK, DC, and T-cell responses [73]. It was also revealed that a low level of IL-17 could lead to MDSC differentiation in vitro, yet, IL-17 could decrease cell proliferation and induce apoptosis in MDSCs [74]. In summary, Th17 cells play a complex role in BC as well, with both pro-tumor and anti-tumor effects. While their pro-tumor properties are primarily associated with the upregulation of angiogenic factors or the secretion of mediators by the other cells leading to Th17 expansion, the anti-tumor properties could result from Th17 interactions with effector immune cells such as Th1 and CTLs.

## 4. IL-25-Producing T-Cells

IL-25-producing cells were recently proposed as a potentiate subset of helper T-cells, Th25, identified by the production of Act1, IL-4, IL-13, and IL-25 (also known as IL-17E). This subset and its main cytokine, IL-25, is commonly involved in response to extracellular pathogens and plays a major role in autoimmune diseases and allergic inflammation [75,76,77]. It is also suggested that Th25 is involved in the processes promoting immunosuppression or tissue repair along with Th2 and M2-macrophages [78]. Nevertheless, defining the role of Th25 cells in cancer is still in the early stages, but some studies have suggested that IL-25, its key cytokine, plays a role in modulating the immune responses to tumors, including BC [79]. IL-25 is a member of the IL-17 family with a unique structure and function. In this connection, it was demonstrated that IL-25, similar to IL-17A, could accelerate the proliferation and survival of tumor cells through the same oncogenic signaling pathways [80]. IL-25 was also shown to have a shared pathway with the epidermal growth factor receptor (EGFR) and rendered TNBC resistant to anti-mitotic and anti-EGFR therapeutic agents [69,80,81]. Bioinformatics analyses also showed similar results in the luminal subtype where high expression of ER was associated with higher expression of IL-25, which in turn suppressed IL-17 signaling and recruited Th17 cells [82]. Similarly, in a transgenic mouse model of spontaneous BC, it was shown that IL-25 promoted the recruitment of type 2 immune cells and facilitated lung metastasis, whereas blocking IL-25 remarkably decreased the type 2 response (Th2, M2-macrophages, and IL-10) in the tumor microenvironment but significantly increased the expression of IL-12 and the activity of CTLs to kill tumor cells [83]. However, it should be noted that in this study and other similar investigations, the function of Th25 cells might be mistakenly misinterpreted as that of Th2 cells due their shared transcription factors, cytokine requirements for development, and similar functions. Reduced tumorigenicity was also observed when the IL-25 receptor, and IL-25RB, were deleted in the drug-resistant BCs [84]. Concordantly, the expression of IL-25 and its receptor was significantly higher in breast tumors compared with the normal samples while it was undetectable in most normal breast tissues [80,83]. However, some studies reported this expression just on tumor-infiltrating CD4^+^ T-cells, and infrequently on tumor cells [83], or could not find IL-25 in the T-cells isolated from the tumoral and adjacent tissues of the patients with different molecular subtypes [69]. Differential expression of IL-25R was also observed in peripheral blood where it was highly expressed in malignant patients and showed direct associations with poor prognoses, i.e., higher grade/stage tumors, and decreased survival [84,85]; however, the serum level as well as mRNA expression of IL-25 in peripheral blood mononuclear cells was remarkably higher in the healthy controls than in the malignant cases [85].

On the other hand, there are some studies introducing IL-25 as one of the endogenous factors secreted by non-malignant mammary epithelial cells or tumor-associated fibroblasts conferring high cytotoxic activity on BC cells without affecting non-malignant mammary cells [86,87,88]. It was proposed that IL-25 differentially induces caspase-mediated apoptosis through the differential expression of its receptor, IL-25R, on poor prognoses tumors rather than on non-malignant cells [84,86,87,88]. In line with these findings, a combination of rIL-25 and IL-17B silencing (siIL-17B) or a single chain against IL-25R could provide a strong inhibition in cancer progression along with decreased VEGF expression, reduced cell viability, and stimulated apoptosis in BC cells [88,89]. Collectively, Th25 seems to be mostly involved in tumor progression through the secretion of IL-25, which leads to the promotion of type 2 immune responses. However, limited studies also suggest an anti-turmeric role for IL-25 by induction of apoptosis in tumors which requires further investigations.

## 5. IL-22-Producing T-Cells

A subset of T-cells, namely Th22, was introduced which mainly produces IL-22 but not any of the other subsets’ related-cytokines, i.e., IFN-γ (Th1), IL-4 (Th2), and IL-17 (Th17) cytokines. These cells produce multiple cytokines including IL-22, IL-26, IL-33, and TNF-α; however, they exert their function mainly through the secretion of IL-22. IL-22 is a member of the IL-10 family, and its expression is stimulated by inflammatory cytokines, i.e., IL-1β, IL-6, IL-21, and IL-23. It binds to IL-22R (a heterodimer of IL-22R1 and IL-10R2) which is selectively expressed by non-immune cells. Besides the production of anti-microbial peptides (β-defensin-2 and β-defensin-3, and peptides of the S100 family), this cytokine induces the proliferation and differentiation of epithelial cells, which contributes to the pathogenesis of some autoimmune diseases and cancers [90,91,92]. Th22 and IL-22 were studied more in gastro-intestinal cancers in which, besides higher frequency of Th22 and IL-22 expression, IL-22 directly promotes the proliferation of colonic epithelial cells and increases their production of nitric oxide synthase and subsequently increases colonic inflammation and carcinogenesis [93]. In BC, however, there are limited studies; Wang et al. demonstrated that the frequency of Th22 increases in tumor tissues compared with para-tumoral and normal breast tissues. Furthermore, in vitro analysis on TNBC cell lines (MDA-MB-231 and MDA-MB-468) also showed that through the activation of STAT3/MAPKs/AKT pathway, IL-22 enhances tumor cell migration and their resistance to paclitaxel chemotherapy in a dose-dependent manner [94]. Our observation also revealed, on average, more than 2% of CD4^+^ lymphocytes in TDLNs of patients with BC produced IL-22; however, the majority of them simultaneously expressed IFN-γ and to a lesser extent, IL-17. The percentage of the Th22 subset (CD4^+^IL-22^+^IFN-γ^−^IL-17^−^) was extremely low (about 0.6%). We observed that the mean percentage of IL-22-producing CD4^+^ cells significantly increased in patients with higher stages and in those with higher numbers of involved nodes. A non-significant increase in the frequency of Th22 lymphocytes was also found in patients with late stages and higher involved nodes [95]. An increased level of IL-22 was also observed in the sera of patients with BC [96]. In tumor tissue, it was observed that IL-22 and IL-22R1 were mostly expressed in tumor cells and to a lesser extent in stromal cells. The IL-22 expression in tumors was a poor prognostic factor for OS, and along with tumor-IL-22R1, was positively associated with the infiltration of CD68-positive TAM, which together displayed the worst prognosis outcomes regarding both OS and RFS [97]. These findings collectively suggest a pro-tumorigenic function for IL-22-producing cells in patients with BC, a concept further supported by in vitro and animal model studies.

Similarly, in the 4T1 mouse model (a model similar to metastatic triple-negative BCs), the level of IL-22 mRNA showed an increase in tumor tissues compared with normal mammary tissues. In addition, exogenous IL-22 increased the proliferation of BC cells in a STAT3/IL-1β/IL-23-dependent manner. Thus, blocking IL-22 activity may lessen the progression of tumors induced by IL-1 and IL-23 [98]. Consistently, Rasé et al. demonstrated that Th22 along with Th17 were detectable very early in the mass and remained until the final day. In the peripherals, when the tumors were well-established, the frequency of Th22 significantly increased over time. Addressing the mechanism underlying Th22 cell recruitment, using 4T1-IL-6-KO mouse mammary carcinoma, they observed that in the absence of IL-6, total CD4^+^ helper cells including Th17 significantly expanded in the tumors, whereas Th22 and MDSC frequencies reduced in all tissues. Accordingly, they concluded that IL-6 might facilitate tumor growth and confer immunotherapy resistance through tumor cell polarization and expansion of the Th22 cell population [99]. Another investigation revealed an increased IL-22 level in the higher stage of breast carcinoma, and its depletion limited tumor invasion and progression and decreased tumor burden. IL-22 deletion was associated with the reduced expression of the transcription factors involved in the EMT [100]. IL-22 also stimulates sphingosine-1-phosphate (S1P) production in mesenchymal stem cells (MSCs) and promotes the chemotactic migration of breast tumor cells toward MSCs, thereby enhancing BC metastasis to bone marrow. Furthermore, IL-22 accelerates this process by increasing the expression of monocyte chemoattractant protein 1 (MCP-1) and MMP-9 activity in MSCs [101]. In addition, it was observed that following the upregulation of IL-22 and HOXB-AS5 (a long non-coding RNA located in *HOX* gene clusters), the PI3K-AKT-mTOR pathway was activated and increased the growth, migration, and invasion of the MDA-MB-231 BC cell-line [102]. Voigt et al. proposed another mechanism in which breast tumor cells induce IL-22 production by memory CD4^+^ T-cells in an IL-1-dependent manner. The IL-1 (β in humans, α in mice) activated the NLRP3 Inflammasome in the tumor microenvironment and induced IL-22 production in various CD4^+^ T-cells including Th22, Th17, and Th1 cells [103]. However, Weber et al. reported a protective role for IL-22 in the BC mouse model as they observed that the treatment of EMT6 cells with IL-22 not only did not induce angiogenesis and apoptosis but also reduced tumor growth and promoted cell cycle arrest through the reduction of ERK1/2 and AKT phosphorylation [104]. Despite this, most studies, including ours, suggest a pro-tumorigenic role for Th22/IL-22 in BC (Figure 2).

## 6. IL-9-Producing T-Cells

IL-9 and IL-9-producing T-cells (termed Th/Tc9), are mainly introduced as pro-inflammatory mediators involved in the pathogenesis of a variety of autoimmune diseases and allergic inflammations. However, it has also been reported that IL-9 can provide a tolerogenic environment, highlighting IL-9 as a pleiotropic cytokine with both positive and negative effects on immune responses [105]. Nevertheless, how IL-9 and Th/Tc9 cells contribute to the immune responses to cancer remains to be elucidated.

Controversial data were reported about the role of Th9/Tc9 in various types of cancer. On the one hand, IL-9 enhances the immunosuppressive function of Tregs, prevents immunological memory formation, and promotes the development of hematological malignancies via its effect as a growth factor [105,106,107]. On the other hand, some studies support the protective role of Th9/Tc9, even superior to Th1/Tc1 subsets, especially in solid tumors like BC [105]. Besides the direct inhibitory effect of IL-9 on tumor cells [108,109], it was shown that IL-9 and IL-9-producing T-cells provide a unique inflammatory environment in tumor tissues, which promotes tumor-specific T-cell responses, particularly CTLs. In addition, it recruits leukocytes, i.e., DCs, into tumor tissues, increases their survival, and enhances antigen-presentation in draining lymph nodes [105,110].

Limited controversial data are available in BC, as well. Through a longitudinal investigation of soluble factors in the sera of BC patients, Carlsson et al. found that patients with metastatic lesions had higher amounts of IL-9 in their serum over time. This finding suggested a relationship between IL-9 and tumor progression, or tumor load [111]. A higher IL-9 level was also observed in both patients’ sera and in the circulating CD4^+^ T-cells than in those of the healthy ones. IL-9-expressing Th9 cells were more abundant in the CCR4^−^CCR6^−^CXCR3^−^ subset and produced an elevated level of IL-10 and IL-21 following activation. These Th9 cells could mediate higher cytotoxicity in CD8^+^ T-cells via IL-9 and IL-21 expression, and IL-9 blocking led to a significant decrease in CD8^+^ T-cells’ cytotoxicity. Accordingly, it could be concluded that Th9 exerts anti-tumor activity in BC at least indirectly by promoting CD8^+^ T-cell inflammation [112]. In another study, IL-9-producing CD8^+^ T-cells were present in some resected BC tumors while IL-9R was almost present on CD8^+^ T-cells in all patients, yet in different degrees. Although there was no association between the expression of IL-9 and its receptor, IL-9R, and the clinical characteristics, IL-9 production in CD8^+^ T-cells from patients with BC was higher than in the healthy controls with more expression in IL-9R^Hi^CD8^+^ T-cells. The IL-9R^Hi^CD8^+^ T-cell subset also exhibited lower expression of inhibitory molecules, i.e., KLRG-1, PD-1, and Tim-3, on one hand, but higher inflammatory mediators, i.e., IL-2 and IL-17, and lower IFN-γ following the activation compared with the IL-9R^Low^ subset. Together, these data indicated an inflammatory response for IL-9-producing CD8^+^ T-cells in BC, and the low expression of PD-1 on IL-9R^Hi^ CD8 ^+^ T-cells may indicate that these cells are more resistant to inhibitory mechanisms in the tumor microenvironment, enabling them to perform effector functions more effectively [113]. Evaluating the role of IL-9 on cancer development in a mouse model of BC (HER2^+^ and TN models) also demonstrated that IL-9 deficiency through neutralizing antibodies or deleting endogenous IL-9 led to priming host tumor-specific T-cells, acquiring immunologic memory, and the early rejection of BC [114]. These results represent IL-9 as an inhibitor of adaptive responses, so blocking IL-9 could be proposed as a therapy that limits tumor growth; however, the exact role of these cells in immune responses to breast tumors and their trafficking pattern need further in vivo and in vitro studies (Figure 2).

## 7. GM-CSF-Producing T-Cells

Another distinct subset of T-cells, named ThGM, has been introduced, which mainly produces GM-CSF but not IFNγ and IL-4 [115]. However, not limited to helper cells, this subset consists of up to 2% of all helper T-cells and is characterized by the expression of various chemokines and chemokine receptors such as CCR10^+^, CCR4^+^, CCR6^+^, and CXCR3 [116]. It has been shown that GM-CSF-producing T-cells play a role in the pathogenesis of various inflammatory and autoimmune diseases [116,117], yet the frequency and function of GM-CSF-producing lymphocytes have not been widely studied in cancers [118]. Nevertheless, GM-CSF is well-known for its immune-modulatory functions as it can exert both suppressive and stimulatory effects on tumor cells [119]. Limited studies on BC also show both pro- and anti-tumorigenic roles for this cytokine or its corresponding T-cell subsets (Figure 1 and Figure 2) [118,120,121]. Accordingly, we observed lower GM-CSF plasma levels in the patients with higher stages of BC than in those with a lower stage of the disease, which could be attributed to GM-CSF suppression following tumor growth and progression [122]. Concordantly, it was shown that in the murine model of BC, GM-CSF inhibited tumor progression and metastasis through their effects on monocytes and the secretion of soluble VEGFR-1, which in turn inactivates the VEGF and blocks angiogenesis [121]. In addition, GM-CSF overexpressing MCF-7 cells exerted more sensitivity to anti-cancer drugs in Chaubey’s study [120].

On the other hand, it is also proposed that higher GM-CSF in the serum is related to the BC metastasis and increased production of GM-CSF in cancer patients (probably due to inflammatory milieu, i.e., in response to TNF-α and LPS), supports angiogenesis, and promotes tumor growth and progression [123,124]. GM-CSF from activated lymphocytes was also observed to increase the expression of PD-L1 on TAMs via an IFN-γ/STAT3 dependent pathway, and provided an immunosuppression [125]. Multiple reports also showed that primary breast tumor cells or mesenchymal-like BCs could aberrantly produce GM-CSF [126,127]. This BC-derived GM-CSF seems to play a pro-tumorigenic role and as an endogenous signal, develops an immunosuppressive microenvironment by inducing plasmacytoid DCs (pDCs)’s activation, which in turn deviates naive CD4^+^ T-cells and macrophages into a regulatory Th2 response and pro-invasive TAM-like phenotype, respectively [128], inducing an EMT/stemness-like invasive phenotype [129] or accumulating Arginase 1 expressing myeloid cells [127]. These conditions were also associated with the more aggressive BC subtypes, metastasis, and reduced survival [126,127,129,130]. In line with these, GM-CSF neutralization significantly reduced tumor growth and metastases probably in part due to the modulation of the tumor microenvironment, the reduction of angiogenesis, and the immunosuppressive cells within the tumor [119]. These discrepancies propose that GM-CSF effects might be source-, dose-, and context-dependent, and further studies are required to clarify the mechanisms by which GM-CSF affects breast tumors. Despite these studies, most observations, in addition to the immunostimulatory effects of GM-CSF in immunotherapies alone or in combination with chemotherapy [131,132], suggest a protective role for this cytokine in the BC tumor setting or its therapeutic potential in the treatment of BC (Figure 1).

## 8. Helper and Regulatory Follicular T-Cells in Breast Carcinoma

Follicular helper T-cells (Tfh) are one of the most prevalent and significant populations of effector T-cells with specialized functions in lymphoid tissues. The expression of CXCR5 enables them to localize within B-cell follicles, where they provide help to B-cells. They also produce IL-21, a potent stimulator of the B-cell differentiation into antibody-forming cells. Tfh cells also express several markers, including the CXCL13 chemokine, the PD-1 and inducible co-stimulator (ICOS) co-stimulatory/inhibitory molecules, and the Bcl6 transcription factor [133,134]. The dysregulated function of Tfh or its related molecules, i.e., ICOS or IL-21, has been reported in certain autoimmune diseases or immunodeficiencies [133]. Few studies have considered the role of these cells in cancer as the origin of follicular lymphoma or other hematologic cancers, as well [135]. However, except for a few recent investigations studying Tfh cells in breast carcinoma, the role of these cells in the immunity against solid tumors has been poorly demonstrated.

In a comprehensive study on infiltrating CD4^+^ T-cells in untreated invasive primary breast tumors, the Gallo group reported, for the first time, the presence of Tfh cells among infiltrating lymphocytes in BC. Most of these CXCL13-producing Tfh cells were in the germinal centers (GCs) of tertiary lymphoid structures (TLS), predominantly located adjacent to the tumor bed, and constituted one of the important components of these structures in the breast tumors [19]. However, through ex vivo functional studies, they later showed signals initiating from ICOS/ICOSL interactions led to Tfh differentiation and migration to the B-cell follicle. It was observed that just a subset of Th1-oriented Tfh with the activated phenotype (PD-1^hi^ICOS^int^) was functional, could produce IFN-γ, provided help for the production of immunoglobulin, induced effector memory B-cells, and was correlated with better disease outcomes [136]. A similar subset was also observed in TDLNs of patients with BC in our previous study with the mean frequency of 2.19 ± 1.59, which showed a positive relationship with both B-cells and Th2 cells, highlighting the collaboration of Tfh cells in configuring antibody responses [22].

Further analysis revealed the association of a Tfh-related gene signature composed of eight genes including *CXCL13* with a greater chance of responding to preoperative chemotherapy and improved survival in patients who have not received treatment [19]. Immunohistochemistry staining also confirmed a correlation between CXCL13 expression and the degree of immune infiltration [19]. The activation of the CXCL13–CXCR5 chemokine axis was previously shown in BC [137]. Although CXCL13 overexpression in BC tissues and increased serum level of this chemokine in patients with metastatic disease suggested a role for CXCL13 in the BC progression [138], the activation of this axis was negatively associated with determinants of a poor prognosis, including axillary node involvement and high histological grade and improved outcome in HER2 overexpressing BC [137]. In addition, the positive correlation of CXCL13 with mesenchymal markers (Vimentin, N-cadherin, Snail, Slug, and MMP9) and negative correlation with E-cadherin implied that the CXCL13–CXCR5 axis could have a role in the regulation of EMT during lymph node metastasis [138,139].

In a complementary study, it has been further demonstrated that the main source of CXCL13 in human BC was CD4^+^ TILs (and to a lesser degree, CD8^+^ TILs), but not follicular DCs. It was proposed that after consuming IL-2 by Tregs and their proliferation at the tumor site, the IL-2 level for effector TILs decreased. Under this condition, some activated CD4^+^ TILs upregulated CXCL13 and were differentiated to CXCL13-producing CXCR5^–^ Tfh (TfhX13). It has been shown that suppressive cytokines (e.g., TGF-β) of Tregs had no effects on the accumulation of these cells. Thus, the frequency of TfhX13 gradually increased at the tumor site, which in turn, directed B-TILs’ migration and subsequently promoted lymphoid structure and GCs’ formation. These cells also regulated the activation and recruitment of CD8^+^ lymphocytes. Since long-lived B-cells and plasma cells are generated in the GCs and play a crucial role in the elimination of tumor residues, the balance between TfhX13 and Treg-TILs seems to be an important issue. In fact, TfhX13 differentiation might be a feedback response that participates in the second round of humoral and cell-mediated immune responses to overcome Treg-mediated immune suppression. However, TfhX13 cells intriguingly expressed considerable levels of PD-1, ICOS, and an intermediate level of intracellular cytotoxic T-lymphocyte-associated protein 4 (iCTLA-4), and accordingly can be considered as an important target for the modulation of the immune system by checkpoint inhibition in BC [136,140]. The higher expression of PD-1 and TIM-3 on Tfh cells was also observed in the peripheral blood of patients with BC. It has been shown that TIM-3^+^ Tfh cells expressed higher levels of PD-1 and were considered to possess an exhausted phenotype as they had lower proliferation, and CXCL13 and IL-21 production. Moreover, B-cells cocultured with these TIM-3^+^ Tfh cells produced less IgM, IgG, and IgA [141].

Collectively, it can be concluded that a combined activated/suppressed TILs profile with higher TLS and GC along with increased Tfh cells in extensively infiltrated BCs suggests that patients with an organized immune response to their tumors, specifically those with detectable Tfh signature, are more likely to respond better to preoperative chemotherapy or have improved postoperative DFS [19].

Besides the specialized T subset with helper function in the follicles, a novel subgroup of Foxp3^+^ regulatory T-cells has been reported recently, which similar to Tfh cells, expresses Bcl6 and enters the follicles by upregulating CXCR5. These cells, designated as follicular regulatory T-cells or TFRs, along with other regulatory cells, seem to play a crucial role in regulating Tfh cells and GC responses and preventing auto-antibody production [142]. The presence and role of this subset in tumor immunity, especially humoral immune responses, have been rarely studied [143,144]. We previously reported a subgroup of CD4^+^Bcl6^+^CXCR5^int/hi^, which were positive for the Foxp3 marker, in TDLNs of patients with BC. This subgroup comprises about one percent of helper cells; however, no significant changes were observed in the frequency of these cells following tumor cells’ infiltration to lymph nodes or progression of the disease [22]. No study has been done on the origin and differentiation of TFR cells in BC, though in a study on human follicular lymphoma, it has been suggested that mesenchymal stromal cells support the viability of TFRs and also mediate differentiation of Tfh to TFR cells through the upregulation of Foxp3 in Tfh cells [145]. These cells with a demethylated *FOXP3* gene could suppress Tfh functionality in a glycoprotein A repetitions predominant (GARP)-associated TGF-β-dependent manner [136]. These cells also showed increased frequency in the peripheral blood of BC patients and functionally increased IL-10 by directly producing IL-10 and indirectly by stimulating the differentiation of IL-10-producing B-cells [146]. A study on Bcl6FC mice, which has a specified deletion in the *BCL6* gene in Foxp3^+^ T-cells and is commonly used as a mouse model for studying TFR, concordantly revealed that TFR cells promote B-cell growth and entry into the dark zone of the GCs through IL-10 production [147]. These findings, collectively, suggest an essential role for TFR cells and their relative balance with Tfh in regulating GC-dependent antibody responses.

## 9. Cytotoxic T-Cells and Their Effector Subsets

Classically, CTLs have been considered the key component of effective anti-tumor immunity [148]. In BC, according to the reports, CD8^+^ lymphocytes are among the most frequent infiltrating subpopulations in the tumor, and those tumors with high infiltration of CD8^+^ T-cells had a better prognosis and survival [148,149]. The gene expression profile of BC stroma also showed that the gene signatures related to CTLs and NK cell activities are predictive of a good outcome with a 5-year survival rate of more than 98% [32]. Concordantly, the frequency of tumor-infiltrating CD8^+^ T-cells showed a negative association with advanced stages and metastasis but a positive correlation with RFS and OS [149,150].

It was also shown that the majority of infiltrating CTLs in tumor beds exhibited effector (CD8^+^CD28^+^), memory (CD45RO^+^), or activated phenotype (CD25^+^, CD69^+^, HLA-DR^+^) and the naive subset (expressing CD45RA and CCR7) compromised the least proportion [148,151,152]. The frequency of CD8^+^CD28^+^CD25^+^ effector cells was found to be negatively correlated with the prevalence of distant metastasis in infiltrative ductal carcinoma of the breast [148]. However, there are also some reports showing the association of CD8^+^ T-cells with survival rate just in some tumor subtypes [153,154], or indicating no obvious association [155] or even an association with poor outcome [26,156].

The discrepancies in the results may be due to the activation status as well as the heterogeneity in the expression profiles of these cells in differential states of anti-tumor immune responses, since similar to CD4^+^ helper lymphocytes, CTLs are also classified into different subsets based on their transcription factors, cytokine profiles, and effector functions, which could dramatically affect the outcome of the host–tumor interaction [157]. In this regard, we observed that the mean expression of IFN-γ in Tc1 lymphocytes in TDLNs was lower in patients with positive nodes and late stages, whereas the frequencies of Tc2 and Tc17 were higher in advanced stages of BC. These differences were more pronounced in patients whose tumor type was infiltrative ductal carcinoma. Higher frequencies of Tc2 and Tc17 were also observed in patients with more involved nodes (N3) compared with the node-negative ones [23]. In this regard, it was shown that in the breast tumor microenvironment, CD8^+^ T-cells are induced to produce IL-17 (Tc17) by TGF-β, which in turn enhances tumor growth directly in tumor-bearing mice and suppresses apoptosis. Consistently, knocking down the receptor of IL-17 increased apoptosis and reduced tumor growth [158]. Albeit later, Tc2 effector subpopulation was also demonstrated to be localized in the tumor site along with Th2 with higher IL-4 and IL-10 production which seemed to be correlated to tumor growth and metastasis in untreated mice [18].

Although there are many reports that CTLs in the tumor microenvironment had an activated phenotype, there is no guarantee that intratumoral T-cells are fully functional [17]. In this connection, the proliferation capacity and IFN-γ production of intratumoral CD8^+^ T-cells were observed to be reduced in response to T-cell receptor stimulation in higher stages [159]. The anti-tumor activity of CTLs was also found to be impaired because of the significant loss of the CD3ζ chain and the CD3-complex dysfunction in the early stage of BC [160]. In addition, T-cell exhaustion could occur due to repetitive and chronic exposure of CTLs to the antigens in the tumor microenvironment, which represents the loss of cytokine production, proliferation, and/or cytolytic activity [161]. In this regard, we observed the higher frequency of TIM-3^+^CD8^+^ cells in TDLNs of patients with high-grade BC. The expression level of TIM-3 in CD8^+^ and CD4^+^ T-cells was also higher in patients with more involved lymph nodes [162]. Similarly, the co-expression of PD-1 and LAG-3 was demonstrated to be associated with exhaustion in CD8^+^ T-cells in patients with TNBC [163]. However, cytokine production, degranulation, and cytotoxic capacity of PD1-expressing CD8^+^ T-cells were reported to be maintained in BC, whereas the same population showed reduced function and exhaustion in melanoma [151,164]. In addition, it was revealed that the tumor microenvironment changes the anti-tumor function of CD8^+^ T-cells through the induction of the CD39 expression (an immunosuppressive ATP ectonucleotidase) and the reduction of effector markers. These CD39^high^CD8^+^ Tregs were only detected in tumor tissues and metastatic lymph nodes but not in the peripheral blood. Upregulation of CD39 was accompanied by the expression of inhibitory receptors and reduced IL-2 and TNF secretion, and tumor growth, indicating CTLs’ exhaustion [165]. Controversially, Tallón de Lara et al. introduced CD39^+^PD-1^+^CD8^+^ T-cells, but not all CD8^+^ T-cells, as a protective population that correlated with DFS [166]. In addition, a population of CD8^+^ T-cells with the suppressive phenotype (CD8^+^CD28^−^) was also detected in the tumor microenvironment [148]. These senescent cells may exert an immunosuppressive function partly through the induction of tolerogenic antigen-presenting cells (APC) [167]. These findings further confirm the considerable heterogeneity of CD8^+^ T-cells in the breast tumor microenvironment and emphasize that several parameters should be considered in the study of CD8^+^ T-cells, including the type and even subtype of cancers, and the expression of immune checkpoint inhibitors or activation markers.

## 10. Memory T-Cells

Memory T-cells are crucial players in the immunity against tumors due to their potential for providing a quick and sustained immune response to the tumors [168]. Hence, several studies showed the importance of CD45RO^+^ memory T-cells in BC prognosis as well. Immunohistochemical evaluations revealed that CD45RO^+^ lymphocytes were the most frequent immune subset in both the periphery and invasive margin of breast tumors indicating previous antigen priming in tumor tissues, while their infiltrations in normal-like tissues adjacent to the tumors were remarkably low [169,170,171]. The frequency of these cells in invasive margins was observed to be strongly higher than in the center of tumors [11]. However, Schnellhardt et al. reported relatively low density for CD45RO^+^ lymphocytes in BC tissues along with poor epithelial infiltration [172]. Nevertheless, the findings collectively showed that higher infiltrations of CD45RO^+^ lymphocytes were significantly associated with smaller tumor size, fewer metastatic lymph nodes and fewer peri-tumoral lymphatic invasions, lower histological grade, and TNM-stage [173,174], although we conversely observed this association with node metastasis, higher histological grade and stages [11]. The older patients (over 50) also had a higher frequency of CD45RO^+^ lymphocytes, consistent with the research on immune system development suggesting an increase in the CD45RO^+^ memory population with age [175]. Immunophenotyping the CD45RO-expressing lymphocytes infiltrating breast tumor tissues revealed that the majority of CD45RO^+^ lymphocytes displayed the CD3 pan T-cells marker (more than 90%), followed by CD8^+^, CD4^+^, and CD16^+^/CD56^+^ NK cells. We also observed that the majority of the NK cells in BC had NKT phenotype expressing CD3 (more than 90%). CD45RO was also rarely expressed on CD14^+^ monocytes and CD11c^+^ DCs (less than 10%), with no expression on CD19^+^ B-cells [unpublished data].

Studies mostly look into the patients generally without considering differences caused by the patients’ molecular subtypes or heterogenicity that exists among memory T-cells. Accordingly, based on the expression of ER, PR, and HER2, we classified the patients into different subgroups: luminal and non-luminal (including HER2-enriched and TNBC) subtypes. Our findings indicated remarkably more infiltrations of CD45RO^+^ lymphocytes in the non-luminal subtypes, with the highest frequency in HER2-enriched tumors, than the luminal type [11]. However, limited studies have investigated CD45RO^+^ lymphocytes in BC with different molecular subtypes [11,176,177]; these observations are in accordance with studies reporting more infiltration of immune cells in non-luminal tumors [178,179]. One of the possible causes of the difference in lymphocytic infiltration among different molecular subtypes was proposed to be the modulatory effect of hormone receptors on immune responses to the tumor and lymphocytic composition in the TME [180,181,182].

In addition to tumor heterogeneity, it is now well-documented that there is a broad diversity among memory T-cells with unique properties within key functional features such as trafficking, localization, effector functions, and durability [183]. In this regard, CD103^+^CD8^+^ T-cells, recognized as tissue-resident memory T-cells (TRM), were observed to be a common effector subset in breast tumors with high expression of effector markers and immunological checkpoint molecules and were linked to improved survival [152]. Immunohistochemical analysis showed a correlation between CD103 positivity and tumor grade, tumor size, and ER/PR status. Additionally, co-expression of CD103^+^/CD8^+^ demonstrated a stronger predictive value and was associated with higher RFS and OS. Therefore, it was suggested that CD103-CD8 co-expression would be a more valuable prognostic marker than CD8 alone [184]. Another independent investigation demonstrated TRM enrichment (defined by CD39, CD69, and CD103 expression) in TNBC samples and its relationship to survival. However, in this study, TRM exhibited an exhausted phenotype, which could be reinvigorated to active form with the addition of immune checkpoint inhibitors [185]. Accordingly, the TRM cell is introduced as a prognostic factor that plays an essential role in BC surveillance and is a key mediator in immunotherapy success. We also assessed memory subsets in draining lymph nodes of patients with BC. We observed that most lymphocytes (both CD4^+^ and CD8^+^ subsets) in draining lymph nodes from patients with BC exhibited a memory phenotype, with the highest frequency for central memory T-cells (TCM) and the lowest for T memory stem cells (TSCM) subsets. Statistical analysis indicated that tumor-positive lymph nodes had higher frequencies of CD4^+^ TSCM and TCM than lymph nodes without tumors. In addition, the frequency of TCM with low expression of CD45RO increased in the advanced stages, while TEM cells decreased [186]. Thus, we hypothesized that in an attempt to provide a pool of memory and effector T-cells, TSCM cells proliferate following long-term exposure to tumor antigens; however, the tumor microenvironment prevents TCM from differentiating into effector cells [186,187]. To check the inhibitory state of lymph nodes, in another study, we assessed the expression of PD-1 and its ligands, as immune checkpoint inhibitors on different memory subsets and found that while PD-1 expression was very low on TSCM, it was highly expressed on TEM and TCM (unpublished data). Although PD-1 was generally considered a hallmark of T-cells’ exhaustion, it was shown that the expression levels of this inhibitory molecule change depending on the stage of the T-cell differentiation [188], suggesting that PD-1 expression on memory T-cells is related to the activation and differentiation status of the subsets rather than their exhaustion. However, more functional studies are needed to elucidate the regulatory role of the PD-1 molecule in memory cell development and function in BC.

## 11. Immune Suppression in Breast Carcinoma: A Role for Regulatory T-Cells

Substantial evidence from functional studies demonstrated that the microenvironment of breast tumors contains a large number of infiltrating leukocytes that do not expand or function normally [17,189]. It is now well-documented that the growth of most invasive carcinomas including BC not only depends on regulatory factors derived from malignant cells but is also affected by those from nearby stromal cells [9,190,191]. In this regard, a large number of studies have indicated that the frequency of regulatory cells increases in the peripheral blood, TDLNs, and more greatly in different tumor tissues including breast adenocarcinomas [22,70,192]. However, Foxp3^+^ regulatory T-cells have low frequency in BC tissues, and their densities and localizations in a large series of patients with primary BC showed a significant association with higher tumor grade, HER2 positivity, ER negativity, and poor prognosis [11,193,194,195]. Infiltration of Foxp3^+^ cells also predicted lymph node metastasis and was associated with a shorter RFS independent of other prognostic factors [193,194,196,197]. The higher frequency of Tregs was also observed in TDLNs of the patients with at least one involved node compared with those with the tumor-free nodes [22]. Interestingly, the proliferation potential of Tregs was shown to be enhanced during disease [159], while we observed that regulatory populations contained a lower percentage of IL-2 and IFNγ-producing cells compared with effector cells [198]. Many studies extend these findings in the animal models and show that Tregs increased in BC and their specific depletion markedly suppresses tumor growth and provokes strong and persistent anti-tumor immune responses [199,200]. The suppressive role of Tregs was further confirmed by their adoptive transfer into the depleted mice that abrogated the immune response [199,200].

Some other studies, however, did not find any significant predictive role for Tregs in BC [11,148] or intriguingly reported that Foxp3 expression in tumor tissue is an independent predictor of better outcomes in HER2^+^ patients [201] or those who received chemotherapy [202]. Besides intrinsic differences among different patients, this discrepancy might be also attributed to the heterogeneity of Foxp3^+^ cells, their functional status, and the expression of different immunosuppressive molecules used to phenotype Tregs. Consistently, phenotypic and transcriptional profiling of the tumor and normal-tissue Tregs exhibited that these cells were Foxp3^+^Helios^+^ cells with high levels of inhibitory molecules (PD-1 and CTLA-4) and were associated with poor prognosis [203]. It is also reported that CCR8 was upregulated in tumor-tissue Tregs and these CCR8^+^ Tregs have high activity and proliferation potential [204]. Controversial reports could also be found regarding CCL22. While there are several studies indicating that CCL22 contributes to the recruitment of Tregs to BC tissues and worse clinical outcomes [205,206], Freier et al. reported that this phenotype correlated with good prognosis. They intriguingly proposed that Tregs confine inflammation and tumor spreading, thereby attenuating nodal involvement [207]. In another study, immunochemistry analysis showed a positive relationship between CCL1 expression, but not CCL22, with Tregs frequency and improved survival in breast tumors [208]. In addition, finding a new subset of Foxp3-expressing CD4^+^ cells with no expression of CD25 increases the Tregs’ complexity. CD25 (IL-2 receptor alpha chain) is commonly considered an indicative marker for both murine and human CD4^+^ Tregs. The presence of this subset has been introduced in different settings including cancer [209]. To the best of our knowledge, it was for the first time that we reported the presence of this subset in draining lymph nodes of patients with BC. This subset showed elevated frequency in node-positive patients with the invasive ductal carcinoma subtype of BC implying an inhibitory role for these cells in breast tumor immunity [22]. However, further evaluations of these cells revealed lower Foxp3 expression compared with the CD25^+^ Tregs [192,198] and lower inflammatory and inhibitory cytokines, i.e., IFNγ, IL-2, IL-17, IL-22, and IL-10, compared with the effectors. We concluded that due to lacking the CD25, these cells probably do not respond to IL-2, and accordingly do not expand and produce effector cytokines. Our results introduced these cells as a heterogeneous exhausted population containing both effector and regulatory cells and/or a subset in the intermediate state [198]. Overall, to clarify the precise role of Tregs in BC, several factors including the heterogeneity of Foxp3^+^ cells and the expression of various immunosuppressive molecules should be considered during studying these cells.

## 12. T-Cell and B-Cell Crosstalk

While the most of focuses are on T-cells in cancer, immunosurveillance is not just limited to T lymphocytes [210]. In fact, the crosstalk of T-cells with other innate and acquired immune cells finally determines the outcome. The interaction of B-cells, the humoral arm of the adaptive immune response, and T-cells becomes more pronounced in GCs where T-cells provide help for B-cells to produce more efficient antibodies [211]. In this regard, Gu-Trantien et al. showed that in BC tumors, inflammatory milieu including Tfh cells directed B-cell migration and subsequently induced lymphoid structure and GCs formation, and finally led to the generation of long-lived B-cells and plasma cells and the elimination of tumor residues [140]. Besides this traditional belief, the crosstalk of B-cells and T-cells is not limited to antibody production but it is a dual and more complex interaction [212].

B-cells constitute up to 40% of TILs in BC tissues and draining nodes [210,213], with dominant memory phenotype (CD24^hi^CD27^+^) in TDLNs [213]. Recent studies showed a strong association between B-cells and different helper and cytotoxic subsets of T-cells. Infiltrated B-cells or a higher ratio of CD20/Foxp3^+^ were also associated with improved clinical prognosis and longer OS [214,215]. B-cells could facilitate tumor regression by presenting antigens to T-cells via MHC I/II, and the recruitment of other immune cells including T-cells to effector sites, secondary and tertiary lymphoid organs [210]. In addition, antibodies bind to tumor antigens and help macrophages and DCs to uptake, process, and present or cross-present them to CD4^+^ and CD8^+^ T-cells, respectively [210]. However, Deola et al. introduced a novel interaction between CTLs and B-cells independent of common antigen presentation, recommending a helper role for B-cells. They showed that engagement of CTLs with bystander B-lymphocytes through CD27/CD70 contact led to the increased proliferation and improved survival of CD8^+^ T-cells [216]. Additionally, B-cells express TNF-α which can provide the co-stimulatory signal for either T effector or Tregs [217]. However, we observed that the frequency of B-cells expressing high levels of TNF-α negatively correlated with the frequency of Foxp3^+^ Tregs in BC draining LNs [217].

On the dark side, it has been shown that B-cells could suppress the anti-tumor function of T-cells through different mechanisms. A subset of B-cells with CD27^hi^CD25^+^ phenotype has been shown to help Treg cell expansion [218]. Consistently, we showed that this subset had a higher frequency in non-metastatic nodes of LN^+^ patients with BC [213]. Another subset of B-cells, IgA^+^PD-L1^+^IL10^+^, impairs the maturation of DCs and the activation of T-cells. In addition, antibodies could bind to FC receptors on myeloid cells and induce their differentiation to MDSCs, and indirectly suppress CD4^+^ and CD8^+^ T-cell responses. B-cells also secret TGF-β and IL-10 that deviate T-cells toward Tregs and suppress T-cell function [210,219]. Based on above, while B-cells are generally known for their anti-tumor effects, primarily through producing antibodies and their interactions with T-cells, specific B-cell subtypes are also able to promote tumor progression.

## 13. Conclusions

Clinical and experimental studies have demonstrated that T-cells play a crucial, yet dual, role in breast tumor development and progression. On the one hand, they suppress breast tumor by destroying tumor cells or inhibiting their growth (Figure 1), and on the other hand, they can facilitate the progression of those tumors that evolved to evade immune surveillance, by inducing the expression of growth factors and regulatory molecules (Figure 2). Despite the presence of many investigations, it is not possible to reach a clear and uniform conclusion about the role of each T-cell subset in the breast tumor microenvironment or its association with BC outcome. In this connection, however, the data were not confirmed by all the investigations; the majority of reports have shown a link between CTLs and Th1 cells and good prognosis, whereas Th2 cells, Tregs, and their related cytokines or molecules correlate with tumor growth and poor prognosis. Information about the exact role of new T-cell subsets such as Th22, Th9, Th25, and ThGM in tumor immunity is still limited and controversial. One of the main reasons for the observed controversies is the use of different markers for the study of one immune cell type. In addition, the classification of T-cells into definite and committed subsets is not simple since it is recently well-appreciated that committed T-cell subsets can be differentiated from other subsets in the inflammatory condition of the tumor microenvironment. Therefore, it is too difficult to divide T-cell subsets into black and white categories in terms of their role in anti-tumor immunity or their association with cancer outcomes. Besides this, the role of T-cells in BC immunity depends on a variety of extrinsic factors, including tumor type or subtype, the stage of the disease, tumor immunogenicity, localization of cells in the tumor tissue and crosstalk with other cells or cytokines.

## Data Availability

Not applicable.
